# Economic Activity and Health Conditions in Adults Aged 65 Years and Older: Findings of the Korean National Longitudinal Study on Aging

**DOI:** 10.3390/healthcare5040063

**Published:** 2017-09-26

**Authors:** Chae-Bong Kim, Seok-Jun Yoon, Jesuk Ko

**Affiliations:** 1 Department of Public Health, Korea University Graduate School, 73 Inchon-ro, Seongbuk-gu, Seoul 02841, Korea; bbp62@nate.com; 2 Department of Preventive Medicine, Korea University College of Medicine, 73 Inchon-ro, Seongbuk-gu, Seoul 02841, Korea; yoonsj02@korea.ac.kr; 3 Department of Healthcare Management, Gwangju University, 277 Hyodeok-ro, Nam-gu, Gwangju 61743, Korea

**Keywords:** economic activity, health, elderly, Korean

## Abstract

The population is aging because lifespans have continued to increase due to developments in modern medical science. The economic activity and health of the elderly are very important factors with reference to the problems of the aged. This cross-sectional study examined the association between the economic activity and health of the elderly. Subjects included 4226 elderly aged over 65 years among the adults who participated in the Fifth Korean National Longitudinal Study on Aging (KLoSA). Basic analysis, cross-analysis, binary logistic regression analysis, and multiple regression analysis were performed to fulfill each research purpose. Male subjects were influenced more by the factors that affect the subjective health of the elderly than females were. Further, subjective health influenced economic activity more than socio-demographic characteristics and health behavior did. Specifically, among the male subjects, the health condition of salaried, self-employed, and unpaid family workers was better as compared to recipients of national health insurance and private health insurance, and unemployed subjects. Preparing for a super-aged society is a worldwide issue. The elderly represent a social participation class that should not be neglected. Therefore, it is necessary to support health promotion policies and increase institutional improvement by reflecting the level of economic activity of the elderly.

## 1. Introduction

### 1.1. Background

Owing to the rapid aging of the society and long-term economic recession, existing social and cultural structures have changed. Consequently, both less-developed and high-income countries are focusing on decreasing the rate of mortality in young people and increasing the rate of the population of the elderly [1,2]. In recent times, the elderly strive for economic independence and aim to lead a healthy life. Additionally, it has been found that the social participation and economic activity of the elderly contributes to improvements in their private life and health [3]. While poor health restricts social activity in the elderly [4], social capital affects their health directly and indirectly [5]. The index of elderly individuals’ economic activity is closely associated with their health. Among retired elderly, work limitation was related to poor health [6]. Further, social and economic level affects the health condition of the elderly [7]. In a study of the health status of health insurance subscribers and recipients of medical care assistance aged 65 years and over in Korea, the latter exhibited twice the incidence of stroke or myocardial infarction as compared to the former [8]. Additionally, another study revealed that jobless elderly were 0.84 times less likely to undergo regular checkups, but the results of these two studies were not statistically significant [9]. Hong et al. reported that disease susceptibility was related to economic activity [8], and elderly persons who had jobs or who participated in social activities with neighbors had optimal subjective health consciousness [10]. Researchers have analyzed social activities and health conditions of the elderly using different methods. Therefore, while one study reported that social and economic levels affect physical health directly and indirectly [11], another study reported that occupation was not associated with health problems [12]. Previous studies have provided the basis for investigating the relationship of demographic characteristics and health behavior or chronic and senile disease incidence with health. However, no study has identified the most influential factor among demographic characteristics, health behavior, and economic activity with reference to the health condition of the elderly. Therefore, the present study aimed to investigate the health condition of the elderly, based on the fact that it determines an individual’s lifestyle, by comparing the effect of demographic characteristics [13], including socio-demographic characteristics, health behaviors, and economic activities. Moreover, this study compared the health conditions of male and female subjects on the basis of the research result that an individual’s lifestyle determined the health condition of male and female groups [13]. According to the results of a systematic review, social status is related to subjective health and well-being [14]. Additionally, one of the most important factors contributing to the health of the elderly was socioeconomic status [15]. Issues of the elderly are important in Korea and in other countries. Therefore, there is substantial research focus on the health of the elderly to sustain their quality of their life and health in the near future.

### 1.2. Study Subjects

This cross-sectional study examined the association between participation in economic activities and health condition in persons aged 65 years and over. Thus, the present study aimed to achieve the following three objectives: (1) to determine the demographic characteristics of workers and non-workers, (2) to investigate the association between economic activity and health condition, and (3) to analyze factors influencing subjective health in males and females.

## 2. Materials and Methods

### 2.1. Research Data

This study used raw data from the Korean National Longitudinal Study on Aging (KLoSA), which was provided by the Korea Labor Institute (approval number 33602). The KLoSA is a panel survey (2006–2014) that investigated the aging process among the Korean elderly aged over 45 years. KLoSA aims to establish data that can measure various aspects of aging, and to construct data to make international comparisons of the aging population possible. Therefore, it will be used as a foundation for establishing and implementing effective social and economic policies in the future. The KLoSA aims to collect data that can measure various aspects of aging, and to construct data which allows an international comparison of the aging population. This data will be used as a foundation for establishing and implementing effective social and economic policies in the future. The sampling framework of the KLoSA followed the computer-assisted personal interviewing (CAPI) method for conducting the population and housing census. Fifth-year (2014) research data were used for the analysis in the present study, which was collected over the 5 months between July and November 2014, using the CAPI. Reports of USA [16], England [17], and Europe [18] were referred to methods of KLoSA’s investigation. The Institutional Review Board’s (IRB) approval of the study was replaced by a research ethics review. The Korea Labor Institute sent a letter of intent to the study of the study and forwarded the letter and notices to the investigation. After the written consent of the interview, it was confirmed that the investigation was conducted.

### 2.2. Outcome Variable

In the study, participants were asked “How do you assess your subjective health status?” Subjective health status was rated by the participants using a five-point scale (ranging from 1 to 5, with 1 indicating very good subjective health and 5 indicating very poor subjective health). Very good health, good health, and usually healthy outcome variables were divided into the good health group and very poor-health and poor-health outcome variables were assigned to the poor-health group.

### 2.3. Materials and Items

The questionnaire items were categorized into items on demographic characteristics, health behavior, and economic activity. Demographic characteristics included gender (men and women), marital status (married and other), educational level (elementary school, middle school, and higher than high school), and area of residence (large city and small city or town). Health behavior included smoking (non-smoker, ex-smoker, and smoker), drinking (non-drinker and drinker), and obesity status (obese, overweight, normal, and underweight). Obesity is generally defined as a person having more fat in their body than a person who is simply overweight, while a person who is overweight is simply heavier than normal [19]. The variables pertaining to health behavior and demographic characteristics were selected based on a previous study [20]. Economic activity included status of coverage by the national health insurance (covered by national health insurance and covered by the medical aid program), status of availing private health insurance, and employment status (salaried worker, self-employed, unpaid family worker, and unemployed). An unpaid family worker is classified as being part of a heterogeneous group of household members, including housewives, elderly persons, and children. They are not paid salaries, but they are a group that engages in economic activities [21]. According to a recent study, elderly unpaid family workers experienced significantly greater odds of reporting subjectively poor physical health [22].

### 2.4. Data Analysis

We performed a cross-tabulation analysis to investigate the characteristics of workers and non-workers on the basis of the study objectives, and tested the significance using a chi-square test. A multiple regression analysis was conducted using a selection method to identify factors influencing subjective health status in workers and non-workers. Furthermore, the chi-square was 5.666 and the significance probability was 0.050 for the Hosmer and Lemeshow test. For all analyses, the significance level was set below 0.05. PASW 20.0 (SPSS Inc., Chicago, IL, USA) was used to conduct the statistical analysis.

## 3. Results

### 3.1. The Outcome Variable

In terms of the outcome variable of subjective health status, 31.6% of the workers (N = 346) and 68.4% of the non-workers (N = 748) reported that they were in a healthy state. In contrast, 19.6% of the workers (N = 615) and 80.4% of the non-workers (N = 2517) reported being in a poor state of health. The two groups showed statistically significant differences in subjective health status (*p* < 0.001) (no table has been presented).

### 3.2. Demographic Characteristics of the Research Subjects

Data on 4226 subjects (workers and non-workers) was included in the analysis. Employment status was statistically correlated with gender (*p* < 0.001), marital status (*p* < 0.001), educational level (p < 0.001), area of residence (*p* < 0.001), smoking status (*p* < 0.001), and obesity status (*p* < 0.001). With reference to the general characteristics, 61.7% of the workers were males and 38.3% were females, and 81.7% of the workers were married. Elementary school graduates accounted for 56.2% of the sample and those with higher than high school education comprised 27.2% of the sample. Further, 26.7% of them resided in a large city and 73.3% lived in a small city or town. Ex-smokers made up 22.5% of the sample, while 17.2% were smokers. Additionally, 40.9% of the workers reported that they consumed alcohol. Finally, 18.5% of the workers had normal weight, and 45.4% were obese (Table 1).

In the non-workers group, 36.4% of the participants were males and 63.6% were females, and 62.2% of the non-workers were married. Elementary school graduates accounted for 63.9% of the sample, and those with education higher than high school comprised 22.3% of the sample. Further, 43.3% of them resided in a large city, while 56.7% resided in a small city or town. Ex-smokers and smokers accounted for 17.6% and 7.8% of the sample, respectively. Finally, 24.1% and 44.0% of the non-workers were normal weight and obese, respectively (Table 1).

### 3.3. Correlation between Economic Activity and Health Condition

Economic activity had a statistically significant correlation with health condition in those who were covered by national health insurance (*p* < 0.001), those who had private health insurance (*p* < 0.001), and those who were employed (*p* < 0.001). Further, 26.9% of those covered by national health insurance and 13.2% of those covered by the medical aid program were in good health. Additionally, 38.7% of those with private health insurance and 24.0% of non-insured participants were in good health. Finally, 38.7% of the salaried participants, 37.1% of those who were self-employed, 25.0% of the unpaid family workers, and 22.9% of the unemployed people were in good health (Table 2).

### 3.4. Subjective Health Status of Males Aged 65 Years and Older

In men, the mean subjective health score of married subjects was 1.697 (1.170–2.461), which was higher than that of unmarried subjects after adjusting for demographic characteristics and health behavior. The mean score of those with education higher than high school was 1.893 (1.490–2.405), which was higher than that of the elementary school graduates. The subjective health status of those with a higher educational status was higher as compared to those of the other group after correcting for demographic characteristics, health behavior, and economic activity. The mean subjective health status of those covered by national health insurance was 2.005 (1.173–3.427), which was higher than that of those covered by the medical aid program. Further, private health insurance applicants showed a higher mean score (1.629 (1.221–2.172)) as compared to those covered by the medical aid program. Finally, the mean subjective health status score of salaried workers was 1.767 (1.256–2.3487), which was higher than that of the unemployed group. Self-employed subjects had a mean score of 1.666 (1.278–2.170), which was higher than that of the unemployed group. This result shows that economic activity and health equity help improve the health status of the elderly (Table 3).

### 3.5. Subjective Health Status of Females Aged 65 Years and Older

In female subjects, the mean score on the subjective health status for high education-level subjects was 2.616 (1.948–3.514), which was higher than that observed in low education-level subjects; therefore, the health status of females shows an increasing trend after adjusting for demographic characteristics and health behavior. In economic activity, the mean score of those covered by national health insurance was 1.865 (1.142–3.045), while private health insurance and employment show a trend of increasing the subjective health status, but not to a statistically significant extent. This result shows that economic activity and economic preparation help improve the health status of the elderly (Table 4).

## 4. Discussion

The subjective health of males was influenced more by economic activity, demographic characteristics, and health behavior. Wilcock et al. argued that, with reference to economic activity, occupation was not fully associated with health problems [12]. Our research result was different from their argument; however, Hong and Kim’s findings corroborated the present findings in that they found that subjective health status was higher in those covered by national health insurance [8]. Furthermore, they found that elderly medical aid beneficiaries had a higher morbidity rate than national health insurance subscribers did [8]. We analyzed the health conditions by gender based on the hypothesis that health outcomes depended on the private lifestyle of the subjects [13]. As a result, engagement in economic activity was found to improve the subjective health status of male subjects. Previous studies revealed that the subjective health status was influenced by social demographic status because it was positively associated with gender, marital status, and socioeconomic status [14,15,23]. It is regarded that the labor, production, and economic consumption of salaried and self-employed individuals positively influences their subjective health status and other factors. Lee argued that health, mediated by economic activities, affects life satisfaction [24], and the authors of another study asserted that it mediates the individual’s social network, including quality of life [8], health behavior, accessibility to medical treatment service, and social participation [25].

Several factors have been found to influence the subjective health status of the elderly. However, the present study found that the variations in health status were caused by economic activities. Kim argued that the health condition was related to income and property ownership, which are indices of economic activity [26], and another study showed the health gap between elderly persons <74 years of different socioeconomic levels [27]. Lee asserted that there was little difference in life satisfaction, but substantial differences in the subjective status depending on the economic participation of the elderly [24]. Further, the perceived subjective health status was higher in the group that was employed and in those who had a large income, and that it promoted friendship with others [10]. It is considered that the elderly with a good socioeconomic status had positive expectations regarding their own health condition [23]. Further, the elderly who participated in economic activities exhibited a 3.97-fold higher tendency to undergo regular checkups as compared to the unemployed elderly [28]. Thus, participation in economic activity may be the main variable that affects the subjective health status of the elderly. On the other hand, the result shows that elderly persons who had no spouse or had a small income experienced more discrimination [29] indicates that there is great pressure on the elderly in terms of sustaining their health condition [10]. Therefore, although it is possible to infer that there is an association between subjective health status and economic activity or the economic level of the elderly, this finding is not sufficient to explain a causal relationship. 

For females in the present study, the subjective health status was significantly associated only with being covered by national health insurance. However, the subjective health status of married females was higher as compared to their counterparts. As a result, it can be assumed that the subjective health status of women is more influenced by their demographic characteristics than by their economic activities. Further, female health was found to be associated with the economic activities and social demographic background of their spouse. Kim argued that the morbidity rate of chronic diseases was higher and subjective health status was lower in females than in males [26]; however, it is unclear if this difference is rooted in their economic activities. It is, therefore, necessary to analyze the differences in health status in women based on their economic activities in future studies. The elderly comprise a socially-active group, and they should not be neglected. Therefore, appropriate employment promotion policies need to be implemented to support the economic activities of the elderly. Economic activities of the elderly are an important factor to satisfy their various desires, including alleviating poverty, improving quality of life, and fostering activities of daily living and health. Kim argued that low income, health condition, and the incidence of geriatric diseases are important factors affecting access to health insurance and medical care assistance [30]. 

## 5. Conclusions

This study was conducted for the purpose of health promotion and social participation of the elderly and the study analyzed the relationship between economic activity and health conditions for aged 65 and older.

In conclusion, elderly Korean males were influenced more than females were by the factors that affect their perceived health. Furthermore, their perceived health would influence their economic activity. It is therefore necessary to consider the health equity of the elderly and to guarantee healthcare services by establishing appropriate policies to address the health inequalities.

## Figures and Tables

**Table 1:** The demographic characteristics and health behavior of the subjects.

Variables	Classification	Total	Workers (N = 346)	Non-Workers (N = 748)	*p*-Value
		N	N (%)	N (%)	
Gender	Man	1783	593 (61.7)	1190 (36.4)	<0.001
	Women	2443	368 (38.3)	2075 (63.6)	
Marital status	Married	2815	785 (81.7)	2030 (62.2)	<0.001
	Other (single and divorce)	1411	176 (18.3)	1235 (37.8)	
Education	Elementary school	2626	540 (56.2)	2086 (63.9)	<0.001
	Middle school	610	160 (16.6)	450 (13.8)	
	Above high school	990	261 (27.2)	729 (22.3)	
Area	Big city (population more than 1,000,000)	1670	257 (26.7)	1413 (43.3)	<0.001
	Small city or town	2556	704 (73.3)	1852 (56.7)	
Smoking (N = 4166)	Non-smoker	2972	570 (60.3)	2402 (74.6)	<0.001
	Ex-smoker	779	213 (22.5)	566 (17.6)	
	Smoker	415	163 (17.2)	252 (7.8)	
Drinking (N = 4063)	Non-drinker	1825	551 (59.1)	1274 (40.7)	<0.001
	Drinker	2238	382 (40.9)	1856 (59.3)	
Obesity (N = 4101)	Obesity	935	174 (18.5)	761 (24.1)	<0.001
	Overweight	1095	304 (32.3)	791 (25.0)	
	Normal	1818	428 (45.4)	1390 (44.0)	
	Underweight	253	36 (3.8)	217 (6.9)	

**Table 2:** Economic activity and health condition.

Variables	Classification	Total	Good Health (N = 1454)	Poor Health (N = 3132)	*p*-Value
		N	N (%)	N (%)	
National health insurance	National health insurance	3922	1054 (26.9)	2868 (73.1)	<0.001
	Medical aid program	304	40 (13.2)	264 (86.8)	
Private health insurance	Insurance policy holder	551	213 (38.7)	338 (61.3)	<0.001
	Not insurance policy holder	3675	881 (24.0)	2794 (76.0)	
Forms of employment	Salary worker	323	125 (38.7)	198 (61.3)	<0.001
	Self-ownership	510	189 (37.1)	321 (62.9)	
	Unpaid family worker	128	32 (25.0)	96 (75.0)	
	Unemployment	3265	748 (22.9)	2517 (77.1)	

**Table 3:** Subjective health status of males aged 65 years and older.

Variables	Classification	Model * (95% CI)	Model ^†^ (95% CI)
Marital status	Other (single and divorce)	1	1
	Marriage	1.697 (1.170–2.461)	1.440 (0.982–2.112)
Education	Elementary school	1	1
	Middle school	1.305 (0.970–1.756)	1.232 (0.910–1.669)
	Above high school	1.893 (1.490–2.405)	1.823 (1.427–2.330)
Area	Big city	1	1
	Small city or town	1.033 (0.832–1.283)	1.123 (0.896–1.408)
Smoking	Smoker	1	1
	Non-smoker	1.131 (0.847–1.511)	1.231 (0.916–1.654)
	Ex-smoker	0.748 (0.564–0.994)	0.825 (0.618–1.103)
Drinking	Drinker	1	1
	Non-drinker	1.095 (0.844–1.421)	1.068 (0.820–1.391)
Obesity	Obesity	1	1
	Overweight	1.357 (1.001–1.838)	1.318 (0.968–1.796)
	Normal	0.895 (0.671–1.193)	0.921 (0.688–1.234)
	Underweight	0.400 (0.224–0.712)	0.454 (0.253–0.814)
National health insurance	Medical aid program	–	1
	National health insurance	–	2.005 (1.173–3.427)
Private health insurance	Not insurance policy holder	–	1
	Insurance policy holder	–	1.629 (1.221–2.172)
Employment	Unemployment	–	1
	Salary worker	–	1.767 (1.256–2.487)
	Self-ownership	–	1.666 (1.278–2.170)
	Unpaid family worker	–	0.738 (0.257–2.120)

* Adjusted for demographic characteristics (gender, marital status, educational level, and area of residence) and health behavior (smoking, drinking, and obesity status). ^†^ Adjusted for demographic characteristics (gender, marital status, educational level, and area of residence), health behavior (smoking, drinking, and obesity status), and economic activity (covered by national health insurance, covered by private health insurance, and employment status).

**Table 4:** Subjective health status of females aged 65 years and older.

Variables	Classification	Model * (95% CI)	Model ^†^ (95% CI)
Marital status	Other (single and divorce)	1	1
	Marriage	1.269 (1.023–1.576)	1.182 (0.944–1.478)
Education	Elementary school	1	1
	Middle school	1.356 (0.992–1.855)	1.310 (0.955–1.796)
	Above high school	2.716 (2.030–3.633)	2.616 (1.948–3.514)
Area	Big city	1	1
	Small city or town	0.800 (0.644–0.994)	0.835 (0.670–1.041)
Smoking	Smoker	1	1
	Non-smoker	1.026 (0.449–2.347)	0.885 (0.383–2.048)
	Ex-smoker	1.188 (0.421–3.354)	1.084 (0.380–3.089)
Drinking	Drinker	1	1
	Non-drinker	0.957 (0.733–1.250)	0.924 (0.705–1.212)
Obesity	Obesity	1	1
	Overweight	1.219 (0.912–1.629)	1.192 (0.890–1.595)
	Normal	1.245 (0.959–1.614)	1.235 (0.951–1.604)
	Underweight	0.629 (0.355–1.114)	0.632 (0.357–1.121)
National health insurance	Medical aid program	–	1
	National health insurance	–	1.865 (1.142–3.045)
Private health insurance	Not insurance policy holder	–	1
	Insurance policy holder	–	1.347 (0.994–1.826)
Employment	Unemployment	–	1
	Salary worker	–	1.114 ( 0.719–1.727)
	Self-ownership	–	1.113 (0.705–1.756)
	Unpaid family worker	–	1.227 (0.763–1.973)

* Adjusted for demographic characteristics (gender, marital status, educational level, and area of residence) and health behavior (smoking, drinking, and obesity status). ^†^ Adjusted for demographic characteristics (gender, marital status, educational level, and area of residence), health behavior (smoking, drinking, and obesity status), and economic activity (covered by national health insurance, covered by private health insurance, and employment status).

## Availability of Data and Materials

Data are available on request from the authors.

## Abbreviations

KLoSA: Korean National Longitudinal Study on Aging; CAPI: computer assisted personal interviewing.
